# The Learning Curve of Surgery of Diabetic Tractional Retinal Detachment—A Retrospective, Comparative Study

**DOI:** 10.3390/medicina59010073

**Published:** 2022-12-29

**Authors:** Ciprian Danielescu, Andreea Dana Moraru, Nicoleta Anton, Madalina-Ioana Bilha, Vlad-Constantin Donica, Diana-Maria Darabus, Mihnea Munteanu, Alin Stefan Stefanescu-Dima

**Affiliations:** 1 Department of Ophthalmology, University of Medicine and Pharmacy “Grigore T. Popa”, Strada Universitatii No. 16, 700115 Iasi, Romania; 2 Doctoral School, University of Medicine and Pharmacy “Grigore T. Popa”, Strada Universitatii No. 16, 700115 Iasi, Romania; 3 Department of Ophthalmology, University of Medicine and Pharmacy “Victor Babes”, Piata Eftimie Murgu, No. 2, 300041 Timisoara, Romania; 4 Department of Ophthalmology, University of Medicine and Pharmacy of Craiova, Strada Petru Rares No. 2-4, 200349 Craiova, Romania

**Keywords:** tractional retinal detachment, pars plana vitrectomy, learning curve

## Abstract

*Background and Objectives*: There are few data in the literature concerning the learning curve of tractional retinal detachment (TRD) surgery. We have analyzed the experience gained by a vitreoretinal surgeon over 10 years. *Materials and Methods*: A retrospective, comparative study of 34 TRD cases operated using 20G instruments between 2008 and 2011 (group A) and 94 cases operated using 23G instruments between 2015 and 2019 (group B). The preoperative characteristics, the type of endotamponade, and the anatomical and functional success were reviewed. *Results*: The group A patients had a significantly higher rate of concomitant vitreous hemorrhage (VH) at presentation (64.7% vs. 37.2%) and of non-macular retinal detachments (52.9% vs. 39.3%). The rate of silicone oil endotamponade was high in both groups (76.4% vs. 68.1%), but in group B 25.5% were left without a tamponade (vs. none in group A). A postoperative anatomical success was obtained in 76.5% of eyes in group A and 84.04% of eyes in group B (where it was improved to 89.3% by reinterventions). The presenting visual acuity (VA) was very low in both groups (0.01 and 0.05, respectively). The proportion of eyes with improved or stabilized VA was 85.3% in group A and 79.8% in group B (statistically non-significant difference). *Conclusions*: The anatomical success rate improves quite slowly with increasing surgeon experience and can be further improved by reinterventions. Visual improvement does not match the rate of anatomical improvement. With increasing experience and self-confidence, the surgeon will approach more difficult cases, a fact that may slow down the increase in surgical success rates.

## 1. Introduction

Diabetic retinopathy (DR) is the most common microvascular complication of diabetes mellitus (DM). It affects 93 million people throughout the world (42.1% of type 1 DM cases and 25.5% of type 2 DM cases) and is the leading cause of new blindness in patients aged 20–74 years in industrialized nations [1,2]. The best way to prevent the development and progression of DR is to keep blood glucose levels under control. Therapy for DR includes laser therapy, anti-VEGF therapy, and corticosteroid therapy [3,4,5].

Its most advanced stage, proliferative diabetic retinopathy (PDR), is estimated to affect 1.4% of all individuals with diabetes [3]. Complications of PDR, including nonclearing vitreous hemorrhage (VH) and TRD, account for 25% of diabetes-related visual loss and are typically managed by pars plana vitrectomy (PPV) [1,6]. Tractional retinal detachment is caused by the contraction of fibrovascular tissue. When TDR involves the fovea, PPV should be performed early to avoid irreversible visual loss, while TRD that does not involve the macula should be closely monitored since it may remain stable [7]. The surgical techniques used during PPV in TRD for the relief of traction include segmentation, delamination, and en bloc dissection. Intraoperative complications include bleeding and the formation of iatrogenic breaks, while postoperative complications such as reproliferations, vitreous hemorrhage, re-detachment, or glaucoma may jeopardize anatomical and functional outcomes. Considering these factors, it is safe to say that PPV for TRD is one of the most difficult of all vitreoretinal surgeries.

While several approaches have been used to study the learning curve of phacoemulsification [8,9,10] and refractive surgery [11,12], few authors have studied the learning curve of PPV, usually for rhegmatogenous retinal detachment surgery [13,14,15,16], and even fewer have assessed the progress in gaining experience and skills of young surgeons attempting TRD surgery [17].

This is a retrospective study comparing the results obtained in phase 1 of the learning curve for TRD surgery with those obtained after a 10 year experience, with all surgeries performed by the same surgeon (in the wider context of the improvements of vitreoretinal techniques and the advent of smaller gauge vitrectomy).

## 2. Materials and Methods

This is a retrospective chart review of the TRD cases operated on by the same surgeon (C.D.) in the Ophthalmology Clinic of “Sf. Spiridon” Hospital, Iasi, Romania, a tertiary care center (at the time the only state hospital clinic in North-East Romania where vitreoretinal surgery was performed). Two time periods were compared: surgeries performed between January 2008 and December 2011 (group A) and surgeries performed between January 2015 and December 2019 (group B) (the surgeon, C.D., began training in vitreoretinal surgery in 2007). In the first period of our study, all surgeries were performed using the 20G Accurus vitreoretinal surgical system (Alcon, Fort Worth, TX, USA) and a wide-angle non-contact viewing system (EIBOS, Haag-Streit Surgical GmbH, Wedel, Germany). In the second period, all surgeries were performed with 23G instruments, using a Constellation surgical system (Alcon, Fort Worth, TX, USA) or a Stellaris PC surgical system (Bausch + Lomb, Bridgewater, NJ, USA), and a BIOM wide-angle non-contact viewing system (OCULUS Surgical, Port St. Lucie, FL, USA).

The following information was collected from the paper-based patient records: age, gender, type of diabetes, diagnosis (TRD with or without macular involvement, association of VH), best corrected visual acuity (BCVA) at the time of hospital admission, type of surgery performed, eventual use of an endotamponade, VA at the first and the latest postoperative visit, and follow-up duration (all patients attended at least the first monthly follow-up visit). Visual acuity was measured using the ETDRS chart and recorded as a decimal. A VA of counting fingers (CF) at 50 cm was approximated as 0.01 decimal, hand motion (HM) was considered the equivalent of 0.005 decimal, and light perception (LP) was considered the equivalent of 0.0001 decimal. For an initial VA below 0.02, gain was considered as an increase of 0.02, while a final VA that remained under 0.02 was considered stable (respectively, vision loss if it decreased to no light perception). For an initial VA between 0.02 and 0.1 decimal, gain was considered an increase of 0.02 (and visual loss was a decrease of 0.02). For an initial VA better than 0.1 decimal, change was considered at least one decimal line, i.e., 0.1.

Being a retrospective study, the need for informed consent for this research was waived by the Hospital Ethics Committee, but a standard informed consent was obtained from all patients before surgery. The research was conducted in accordance with the principles of the Declaration of Helsinki.

The statistical analysis included the chi-squared test (comparing two proportions between the groups) performed with the MedCalc statistical software (MedCalc Software Ltd., Belgium). A *p* value of 0.05 was considered the threshold for statistical significance.

## 3. Results

The main data collected from the two groups are presented in Table 1. Group A (patients operated on between 2008 and 2011) included thirty-four patients, of whom nine had type 1 and twenty-five had type 2 diabetes mellitus. Group B (patients operated between 2015 and 2019) included 94 patients, consisting of 10 with type 1 and 84 with type 2 diabetes. There was a significant difference in the proportion of patients who had VH at the time of presentation: 64.7% in group A versus 37.2% in group B, *p* = 0.01.

The 34 patients in group A with TRD accounted for 4.02% of the 845 vitreoretinal surgeries performed by the surgeon between 2008 and 2011. The 94 group B patients with TRD accounted for 4.2% of the 2188 vitreoretinal surgeries performed by the surgeon between 2015 and 2019.

The number of vitrectomies performed for complications of diabetes (VH, TRD, and macular edema with posterior hyaloid traction) in groups A and B was 186 and 392, respectively, accounting for 22.1% and 17.9%, respectively, of the total number of vitreoretinal surgeries performed by C.D. during the two study time intervals.

Non-macular TRD was more common in group A patients (macula-on TRD being found in 52.0% of group A versus 39.3% of group B patients). The proportions were reversed postoperatively, with the percentage of eyes with an attached macula (after the resorption of air/gas and the extraction of silicone oil) being smaller in group A (76.5%) than in group B (84.04%). However, the differences between groups, both preoperatively (*p* = 0.24) and postoperatively (*p* = 0.47), were not statistically significant.

Of the fifteen group B patients in whom surgical macular reattachment had failed, 60% (nine patients) agreed to undergo a reintervention. Techniques such as bimanual dissection of membranes with vitreoretinal forceps and scissors under chandelier illumination and silicone oil injection were used, allowing further attachment of the retina in five cases and thus increasing the final anatomical success rate of group B to 89.3%.

A large fraction of eyes have received a silicone oil endotamponade: 76.4% in group A and 68.1% in group B. However, there was a significant trend towards no endotamponade at the end of the first surgery: 25.5% in group B compared to none in group A.

There was a significant increase in postoperative VA in both groups. In group A, the average preoperative VA was very low: 0.01 ± 0.01 (range 0.0001–0.02), and the postoperative VA increased to 0.12 ± 0.21 (range 0–0.4), *p* = 0.02. In group B, preoperative VA was on average 0.05 ± 0.3 (range 0.0001–0.6) and it increased to 0.19 ± 0.41 postoperatively (range 0.0001–0.8), *p* = 0.01. However, no significant differences between groups were found in the changes in visual acuity after surgery.

A slightly larger proportion of eyes in group B patients have gained VA after surgery (63.8%, compared to 58.8% in group A). The percentage of eyes with stable VA was higher in group A (26.4% compared to 15.9% in group B).

In group A, there were 16 eyes with TRD involving the macular area, of which eight (50%) had an attached macula at the final visit. In group B, 57 eyes initially had macula-off TRD, and in 42 (73.6%), retinal reattachment was successful. The proportion of eyes with initial macular TRD that demonstrated macular attachment at the final visit did not differ significantly between groups A and B, *p* = 0.13.

## 4. Discussion

The interventions aimed at preventing DR in diabetic patients, such as lifestyle changes and therapy to keep blood glucose levels under control, are managed by the diabetes specialist working together with each patient. Fenofibrate is an oral drug with promising results in preventing or delaying DR progression [2]. Panretinal photocoagulation (PRP) was for many decades the mainstay of treatment for the prevention and therapy of early forms of PDR. However, recent results from the Diabetic Retinopathy Clinical Research Network have documented the efficacy of anti-VEGF injections in the treatment of PDR (protocol S) and moderate-to-severe non-proliferative DR (protocol W) [4,5]. In our country, PRP is still the standard treatment for severe non-proliferative DR and PDR. Aflibercept is covered by our National Health Insurance for the therapy of diabetic macular edema; however, until now it did not receive approval for use in PDR.

In a large Royal College of Ophthalmologists’ National Database study, 6% of all vitreoretinal surgical procedures were performed for diabetes complications [18]. The percentage of vitrectomies performed for diabetes complications by the surgeon C.D. in our study was approximately triple that observed in the UK, which could be attributed to poorer management of diabetes mellitus in our population and also to a relatively high rate of failure to diagnose diabetic retinopathy and perform panretinal photocoagulation when the proliferative stage is attained, a problem that has been of concern for a long time [19,20].

The proportion of TRDs in the total number of vitrectomies performed throughout the study period was remarkably stable, indicating that this may be the true proportion of TRDs in the entire population of patients that needed vitreoretinal surgery.

While the initial VA in the two groups was comparable, the proportion of eyes with TRD and VH at presentation was significantly higher in group A. It has been demonstrated that patients who present with TRD and VH have a better visual prognosis than those who need surgery for TRD without VH (presumably with a VA decrease caused by a detached macula) [21,22]. Additionally, a larger number of patients in group A presented with extramacular TRD. These findings indicate that the surgeon has dealt with more difficult cases during the last 5 years covered by this study. The ability to approach more difficult cases comes with experience, and we believe it is a characteristic of the typical learning curve.

In previous retrospective reviews of rhegmatogenous retinal detachment (RRD) surgeries performed by beginner surgeons, the primary success rates were 70% [14], 75% [23], and 80% [13], and they improved and then stabilized after about 200 operations [13] or 2 years [14]. In another study, the learning effect was reduced by half after about 500 vitreoretinal surgeries [24]. Of course, there are differences in learning curves among surgeons, and it was argued that “volume alone does not account for skill levels” [13].

In the only paper dealing with the results of TRD surgeries carried out by vitreoretinal fellows we could find, Rahimy et al. reported anatomical success rates of 85.3% and 96.4%, respectively, after surgeries performed by first-year and second-year fellows, while the percentage of eyes with stable or improved VA was 71% [17]. The successful reattachment rate in our group A was 76.5%, and it increased to 89.3% in our group B, comparable to the results presented by Rahimy et al. Since the presenting VA was very low in our group A, 85.2% of the time it remained unchanged or improved after surgery.

In the study by Rahimi et al., the rate of secondary RRD (i.e., RRD that appeared following TRD surgery) was quite high (17.7%), and second-year fellows had a significantly lower rate of RRDs than the first-year fellows [17]. In our group B, 15.96% had a detached macula after the first surgery. In our study, the anatomical success rate had increased with time (even if not statistically significant, probably due to the small number of cases in the two groups). It became clearer when we looked at the anatomical success rate in eyes with macular TRD at the presentation: we were able to reattach the macula in half of such eyes in group A and three-quarters of such eyes in group B.

Another interesting observation is that in group A, there was no case left without endotamponade at the end of surgery (all eyes received silicone oil, air, or a non-expansile mixture of C2F6 with air). Almost a quarter of the group B patients did not receive an endotamponade, suggesting an increased surgeon’s self-confidence and the absence of iatrogenic breaks at the end of the procedure (however, 68% received a silicone oil endotamponade, a high percentage when compared with the literature data). In a large UK database study by Jackson et al., 57.6% of diabetic vitrectomies with delamination required an intravitreal tamponade (only 11.17% received silicone oil endotamponade) [18]. This level of excellence obtained by experienced UK surgeons is a benchmark toward which we must strive. In an interventional study on 84 eyes with TRD, Qamar et al. performed PPV without endotamponade and obtained retinal reattachment in 92.8% of cases [25]. However, these were not consecutive cases, and eyes with iatrogenic breaks were simply excluded from the series.

The obvious goal of TRD surgery is to relieve all tractions without creating breaks; however, for the beginner surgeon, the use of oil endotamponade is a welcome aid and may stabilize the retina even if some breaks were created (but only if the tractions have been thoroughly relieved). It also may offer faster VA recovery, which is much needed for patients who, by and large, presented with very poor initial VA. In the large majority of cases, we have been able to safely remove the silicone oil in due time and potential intraocular hypertension has been treated with antiglaucoma topical medication.

When the visual results were analyzed, we found a small improvement in the proportion of eyes with VA gain after surgery in group B patients compared to group A. The percentage of eyes with stable VA was higher in group A. In a retrospective study of TRDs operated by Storey et al., VA improved in 56.3% of cases and remained stable in 23.8% of cases, remarkably similar to our study [26].

In fact, the percentage of eyes with improved or stabilized VA after surgery was slightly higher in the first study period than in the second, without reaching statistical significance. One must take into account that two thirds of group B eyes present with macular detachment, which carries a worse visual prognosis. We must point out that the presenting VAs were very low in both our study groups, with an average VA of 0.01 (decimal) in group A and 0.05 in group B. In a retrospective study of vitrectomy for chronic diabetic tractional macular detachment, 43% of eyes had preoperative VA > 0.025, and that proportion improved to 66.7% at the final follow-up [27]. Poor preoperative VA was obviously a risk factor for worse visual results after surgery [27,28].

For the 2008–2011 period, we were unable to reliably establish the subsequent management of patients without an attached macula after the first surgery (we did not attempt to reoperate due to the obvious difficulty of such reinterventions for a beginner surgeon). At that time, the patients did not have an option for another vitreoretinal surgeon in the state hospitals in our region. We know that some of them underwent reintervention in a private practice, while others went to the ophthalmology hospital in our capital city, Bucharest. However, due to socioeconomic factors, some patients did not pursue another attempt at vitreoretinal surgery. For the second study period, between 2015 and 2019, nine of fifteen eyes with initial anatomical failure underwent reoperation by the same surgeon, and the macula was attached in five of them. This has raised the anatomical success rate at the end of the follow-up period to 89.3% in group B.

Due to our study being a retrospective one, we were not able to determine the progression of lens opacities in all vitrectomized eyes. Simultaneous PPV and phacoemulsification is a safe and effective procedure [29]; however, our approach was the first surgery to be aimed at repairing retinal detachment alone. Interestingly, it seems that the interval between PPV and subsequent cataract extraction is significantly longer for eyes operated by experienced surgeons [30].

While 89.3% of our group B patients had an attached retina at the end of the study, 20.2% had some VA loss. This particularly frustrating situation may be attributable to macular ischemia, associated with disorganization of the retinal inner layers and ellipsoid zone loss [27,31], persistent submacular fluid that may take up to one year to absorb [32,33], the preoperative duration of TRD with subsequent loss of photoreceptors, optic nerve ischemia, or advanced secondary glaucoma.

As this surgeon gained experience in the larger context of the evolution of vitreoretinal instruments, it is difficult to quantify the role of decreasing incision size from 20 G to 23 G (nowadays we are performing all these surgeries in 25 G). It is widely recognized that smaller gauge vitreous cutters allow for safer cutting of fibrovascular preretinal membranes, working closer to the retina.

The obvious weakness of this study is its retrospective nature. A possible strength is the fact that it documents the learning curve of a single surgeon in a particular healthcare setting with a high proportion of diabetic vitrectomies compared to the total number of PPVs. A prospective study on the learning curve of this difficult surgery would be difficult to plan, given the relative scarcity of TRDs in the total number of vitreoretinal cases.

## 5. Conclusions

We have confirmed the data reported in the literature on the results of TRD surgeries performed by beginner surgeons. The anatomical success rate increases quite slowly with the surgeon’s experience and can be further improved by reinterventions. Visual improvement does not match the anatomical improvement rate. With experience and increased self-confidence, the surgeon will approach more difficult cases, and this may slow the increase in the surgical success rate.

## Figures and Tables

**Table 1:** Comparison between group A (patients operated between 2008 and 2011) and group B (patients operated between 2015 and 2019).

	Group A (2008–2011)	Group B (2015–2019)	*p*-Value
Number of patients	34	94	
Gender	16 women/18 men	45 women/49 men	
Age (years)	51.7 (range 26–62)	57.02 (range 22–77)	
Type 1/Type 2 diabetes	9/25	10/84	
Preoperative macula-on	18 (52.9%)	37 (39.3%)	0.241
Preoperative VH	22 (64.7%)	35 (37.2%)	0.01
Postoperative macula-on	26 (76.5%)	79 (84.04%)	0.47
Final macula-on (after reintervention)	Not applicable	84 (89.3%)	
Follow-up in months	14 (range 1.5–84)	9.7 (range 1.5–51)	
Endotamponade			
-Silicone oil	76.4%	68.1%	0.49
-Air/gas	23.5%	6.3%	0.01
-no tamponade	0	25.5%	0.002
Percentage of eyes with VA gain	58.8%	63.8%	0.97
Percentage of eyes with stable VA	26.4%	15.9%	0.64
Percentage of eyes with VA loss	14.7%	20.2%	0.65
Preoperative VA (decimal)	0.01 (range 0.0001–0.02)	0.05 (range 0.0001–0.6)	0.27
Final VA (decimal)	0.12 (range 0–0.4)	0.19 (range 0.0001–0.8)	0.51

## Data Availability

Data is contained within the article.
